# Impact of early enteral versus parenteral nutrition on mortality in patients requiring mechanical ventilation and catecholamines: study protocol for a randomized controlled trial (NUTRIREA-2)

**DOI:** 10.1186/1745-6215-15-507

**Published:** 2014-12-23

**Authors:** Laurent Brisard, Amélie Le Gouge, Jean-Baptiste Lascarrou, Hervé Dupont, Pierre Asfar, Michel Sirodot, Gael Piton, Hoang-Nam Bui, Olivier Gontier, Ali Ait Hssain, Stéphane Gaudry, Jean-Philippe Rigaud, Jean-Pierre Quenot, Virginie Maxime, Carole Schwebel, Didier Thévenin, Saad Nseir, Erika Parmentier, Ahmed El Kalioubie, Mercé Jourdain, Véronique Leray, Nathalie Rolin, Frédéric Bellec, Vincent Das, Frédérique Ganster, Christophe Guitton, Karim Asehnoune, Anne Bretagnol, Nadia Anguel, Jean-Paul Mira, Emmanuel Canet, Bertrand Guidet, Michel Djibre, Benoit Misset, René Robert, Frédéric Martino, Philippe Letocart, Daniel Silva, Michael Darmon, Vlad Botoc, Jean Etienne Herbrecht, Ferhat Meziani, Jérôme Devaquet, Emmanuelle Mercier, Jack Richecoeur, Stéphanie Martin, Emilie Gréau, Bruno Giraudeau, Jean Reignier

**Affiliations:** Surgical Intensive Care Unit, Laënnec Hospital, University Hospital, Nantes, F-44093 France; UPRES EA-3826, Clinical and Experimental Therapies for Infections, University of Nantes, Nantes, France; INSERM CIC 1415, CHRU de Tours, Tours, France; Medical-Surgical Intensive Care Unit, District Hospital Center, Les Oudairies, La Roche sur Yon, F-85025 France; Clinical Research Unit, District Hospital Center, La Roche sur Yon, F-85025 France; Medical-Surgical Intensive Care Unit, University Hospital, Amiens, F-80054 France; Medical Intensive Care Unit, University Hospital, Angers, F-49933 France; Medical-Surgical Intensive Care Unit, District Hospital Center, Pringy, F-74374 France; Medical Intensive Care Unit, University Hospital, Besançon, F-25030 France; Medical Intensive Care Unit, University Hospital, Bordeaux, F-33076 France; Medical-Surgical Intensive Care Unit, District Hospital Center, Chartres, F-28018 France; Medical Intensive Care Unit, University Hospital, Clermont-Ferrand, F-63003 France; Medical-Surgical Intensive Care Unit, Louis Mourier University Hospital, AP-HP, Colombes, F-92700 France; Paris Diderot University, Sorbonne Paris Cité, ECEVE, UMRS 1123, F-75010 Paris, France; INSERM, ECEVE, U1123, F-75010 Paris, France; Medical-Surgical Intensive Care Unit, Hospital Center, Dieppe, F-76202 France; Medical Intensive Care Unit, University Hospital, Dijon, F-21079 France; Centre de Recherche Lipides, Nutrition, Cancer U866, INSERM, Dijon, F-21079 France; Medical Intensive Care Unit, Raymond Poincaré Hospital, University Hospital, Garches, F-92380 France; Medical Intensive Care Unit, University Hospital, Grenoble, F-38043 France; Medical-Surgical Intensive Care Unit, District Hospital Center, Lens, F-62307 France; Medical-Surgical Intensive Care Unit, University Hospital, Lille, F-59000 France; Medical Intensive Care Unit, University Hospital, Lyon, F-69004 France; Medical Intensive Care Unit, District Hospital Center, Melun, F-77000 France; Medical-Surgical Intensive Care Unit, District Hospital Center, Montauban, F-82013 France; Medical-Surgical Intensive Care Unit, Andre Gregoire District Hospital Center, Montreuil, F-93105 France; Medical Intensive Care Unit, District Hospital Center, Mulhouse, F-68100 France; Medical Intensive Care Unit, University Hospital, Hotel Dieu Hospital, Nantes, F-44093 France; Surgical Intensive Care Unit, University Hospital, Hotel Dieu Hospital, Nantes, F-44093 France; Medical-Surgical Intensive Care Unit, District Hospital Center, Orléans, F-45067 France; Medical Intensive Care Unit, Kremlin-Bicêtre Hospital, University Hospital, Paris, F-94275 France; Medical Intensive Care Unit, Cochin Hospital, University Hospital, Paris, F-75014 France; Medical Intensive Care Unit, St Louis Hospital, University Hospital, Paris, F-75010 France; Medical Intensive Care Unit, St Antoine Hospital, University Hospital, Paris, F-75571 France; Medical-Surgical Intensive Care Unit, Tenon Hospital, University Hospital, Paris, F-75020 France; Medical Intensive Care Unit, St Joseph Hospital, University Hospital, Paris, F-75014 France; Medical Intensive Care Unit, University Hospital, Poitiers, F-86021 France; Medical-Surgical Intensive Care Unit, University Hospital, Pointe à Pitre, F-97159 France; Medical-Surgical Intensive Care Unit, District Hospital Center, Rodez, F-12000 France; Medical-Surgical Intensive Care Unit, District Hospital Center, St Denis, F-93200 France; Medical-Surgical Intensive Care Unit, University Hospital, St Etienne, F-42055 France; Medical-Surgical Intensive Care Unit, District Hospital Center, St Malo, F-35400 France; Medical Intensive Care Unit, Hautepierre Hospital, University Hospital, Strasbourg, F-67098 France; Medical Intensive Care Unit, Nouvel Hopital Civil, University Hospital, Strasbourg, F-67091 France; Medical-Surgical Intensive Care Unit, Foch-Suresnes Hospital, University Hospital, Suresnes, F-92150 France; Medical Intensive Care Unit, Bretonneau Hospital, University Hospital, Tours, F-37044 France; Medical Intensive Care Unit, District Hospital Center, Beauvais, F-60000 France; Université François-Rabelais de Tours, PRES Centre-Val de Loire Université, Tours, France

**Keywords:** Critical illness, Enteral nutrition, Mechanical ventilation, Mortality, Nosocomial infection, Parenteral nutrition, Shock, Vasoactive drugs

## Abstract

**Background:**

Nutritional support is crucial to the management of patients receiving invasive mechanical ventilation (IMV) and the most commonly prescribed treatment in intensive care units (ICUs). International guidelines consistently indicate that enteral nutrition (EN) should be preferred over parenteral nutrition (PN) whenever possible and started as early as possible. However, no adequately designed study has evaluated whether a specific nutritional modality is associated with decreased mortality. The primary goal of this trial is to assess the hypothesis that early first-line EN, as compared to early first-line PN, decreases day 28 all-cause mortality in patients receiving IMV and vasoactive drugs for shock.

**Methods/Design:**

The NUTRIREA-2 study is a multicenter, open-label, parallel-group, randomized controlled trial comparing early PN versus early EN in critically ill patients requiring IMV for an expected duration of at least 48 hours, combined with vasoactive drugs, for shock. Patients will be allocated at random to first-line PN for at least 72 hours or to first-line EN. In both groups, nutritional support will be started within 24 hours after IMV initiation. Calorie targets will be 20 to 25 kcal/kg/day during the first week, then 25 to 30 kcal/kg/day thereafter. Patients receiving PN may be switched to EN after at least 72 hours in the event of shock resolution (no vasoactive drugs for 24 consecutive hours and arterial lactic acid level below 2 mmol/L). On day 7, all patients receiving PN and having no contraindications to EN will be switched to EN. In both groups, supplemental PN may be added to EN after day 7 in patients with persistent intolerance to EN and inadequate calorie intake. We plan to recruit 2,854 patients at 44 participating ICUs.

**Discussion:**

The NUTRIREA-2 study is the first large randomized controlled trial designed to assess the hypothesis that early EN improves survival compared to early PN in ICU patients. Enrollment started on 22 March 2013 and is expected to end in November 2015.

**Trial registration:**

ClinicalTrials.gov Identifier: NCT01802099 (registered 27 February 2013)

## Background

Nutritional support is a key component of the life-sustaining strategies used on an everyday basis in intensive care units (ICUs) to combat the adverse effects of critical illnesses. The latest international guidelines recommend enteral nutrition (EN) as opposed to parenteral nutrition (PN) as the first-line route for nutritional support [1–4]. EN has documented beneficial effects on gastrointestinal mucosa integrity, wound healing, immune function and response to tissue damage [5–7]. EN may contribute to diminishing nosocomial infection rates, length of stay and health-care costs [8–11]. Early EN initiation (within 24 to 48 hours after ICU admission) may enhance these beneficial effects and decrease mortality rates [12–15]. EN initiation within 48 hours after invasive mechanical ventilation (IMV) has been reported to improve outcomes of patients with multiple organ failure [12, 14]. Despite this evidence base, substantial gaps persist between everyday practice and clinical guidelines about nutritional support. EN is frequently delayed and PN used in patients who are eligible for EN [16–18]. At least two factors may explain the current underuse of EN—namely, legitimate or exaggerated concerns about complications related to EN and the absence of sound scientific evidence that one route is superior to the other [19].

The most common complication of early EN is upper gastrointestinal intolerance, which occurs in 30% to 70% of ICU patients [20]. Upper gastrointestinal intolerance manifests as gastric hypokinesia responsible for an increase in the residual gastric volume, which in turn is believed to increase the risk of gastroesophageal reflux, aspiration and nosocomial pneumonia [21–28]. The sequence of incomplete gastric emptying, gastroesophageal reflux, aspiration and nosocomial pneumonia is a constant source of concern for health-care workers. Ventilator-associated pneumonia (VAP) is a major complication of IMV; it is seen in 8% to 27% of patients and is responsible for longer ICU stays and increased mortality in some patients [29]. The measure most commonly used to prevent or decrease the risk of upper gastrointestinal intolerance is discontinuation or substantial reduction of the enteral feed flow rate. However, this measure decreases energy and nutrient intake below what is required to meet the needs of the patient. A negative energy balance is associated with undernutrition, which in turn correlates with higher rates of infection and death in the most severely ill patients [30–35]. Concern about VAP and undernutrition related to EN intolerance is a major deterrent to the use of early EN [36–45]. EN has also been associated with an increased risk of gut ischemia in critically ill patients with shock [46–49]. Gut ischemia may result in necrotizing enterocolitis, which is fatal in 70% to 100% of cases [50, 51]. Factors involved in gut ischemia include impaired splanchnic blood flow, vasoactive drug use, preexistent arterial disease and specific clinical conditions such as renal failure, heart surgery, multiple trauma, acute respiratory distress syndrome, septic shock, cardiogenic shock and fluid overload [49, 52, 53]. Whether EN contributes to the development of gut ischemia remains controversial. Experimental studies have demonstrated better preservation of gastrointestinal mucosal integrity with EN [54, 55]. However, splanchnic blood flow has been found to increase by 50% with EN and decrease by over 60% with PN, suggesting a risk of inadequate splanchnic oxygen supply in the face of high demand in patients with shock who are receiving EN [46]. Guidelines recommend postponing EN in patients with shock until full resuscitation with achievement of hemodynamic stability [2, 3]. This delay in EN delivery may be associated with calorie deficiencies and adverse outcomes in patients with severe critical illnesses [30, 34]. These considerations explain why clinicians perceive PN as the safest means of delivering adequate energy and protein to patients at high risk for intolerance to EN or gut ischemia.

Another contributor to the current underuse of early EN in ICU patients may be the reliance of international guidelines on studies that have provided low-level evidence and on meta-analyses whose results are conflicting [1, 13, 15]. Most meta-analyses comparing EN and PN have shown no effect on mortality, but have indicated that EN, compared to PN, was associated with decreases in nosocomial infections, hospital and ICU lengths of stay and health-care costs [9, 10, 56–58]. A meta-analysis confined to trials involving an intention-to-treat analysis (9 of 465 trials) demonstrated higher mortality with EN than with PN [59]. However, all the available meta-analyses included highly heterogeneous studies that were done in small numbers of patients and were focused on surgical patients and/or on patients who did not require ventilatory support or who had critical illnesses of limited severity. These characteristics preclude drawing definitive conclusions about the respective effects of EN and PN in critically ill patients. Many authors have emphasized the need for well-designed, adequately powered, randomized trials in uniform populations of ICU patients with severe critical illnesses associated with a high risk of death and/or complications, in whom optimal nutritional support raises the greatest challenges but is likely to have the greatest impact on patient outcomes [59–61].

### Study rationale

Patients with shock requiring both IMV and vasoactive drugs are at high risk for complications and death. Given their vulnerability to EN intolerance and gut ischemia, guidelines indicate that EN should be postponed until hemodynamic stability is restored [2, 3]. These patients consequently receive either delayed EN or initial PN, although they may have a high likelihood of survival benefits from early EN compared to PN, according to several observational [12, 14, 17]. No adequately designed study has compared early EN to early PN in this population. The primary goal of the present trial is to assess the hypothesis that early first-line EN decreases day 28 all-cause mortality in these patients compared to early first-line PN.

## Methods/Design

### Design and setting

NUTRIREA-2 is a multicenter, open-label, parallel-group randomized controlled trial in patients receiving IMV and vasoactive drugs for shock. They will be allocated to early first-line EN or early first-line PN in the acute phase of ICU management.

### Ethical aspects

The study protocol and patient information documents were approved by the ethics committee of the French Society for Intensive Care Medicine (Société de Réanimation de Langue Française (SRLF) approval CE SRLF 11-340) and by the competent French authorities (Comité de Protection des Personnes de Poitiers registration 2011-A01483-38 (approved on 26 January 2012)). According to French law, as the strategies used in both study arms are classified as standard care, patient consent is not required; however, the patients or their next of kin must be informed about the study before enrollment. Before study enrollment, all patients or their next of kin will confirm in writing that they have received this information.

### Participating units

Of the 44 French ICUs participating in the study, 28 are in university hospitals. All participating ICU staff members have received training in the study procedures and protocols for providing nutritional support and managing EN intolerance.

### Study population

Eligible patients are adults (≥18 years of age) admitted to the study ICUs who are expected to require IMV for longer than 48 hours, are being treated with a vasoactive drug (adrenaline, dobutamine or noradrenaline) via a central venous catheter and are eligible for nutritional support started within 24 hours after endotracheal intubation (or within 24 hours after ICU admission if intubation occurred before ICU admission).

Exclusion criteria are IMV started more than 24 hours after endotracheal intubation or ICU admission; surgery on the gastrointestinal tract within the past month; history of gastrectomy, esophagectomy, duodenopancreatectomy, bypass surgery, gastric banding or short bowel syndrome; gastrostomy or jejunostomy; specific nutritional needs, such as preexisting long-term home-based EN or PN; active gastrointestinal bleeding; treatment limitation decisions; adult under guardianship; pregnancy; breastfeeding; current inclusion in a randomized trial designed to compare EN and PN; and/or contraindication to PN (known hypersensitivity to egg or soybean proteins or to another component, inborn error in amino acid metabolism or severe familial dyslipidemia affecting triglyceride levels).

All patients receiving IMV and vasoactive drugs for shock within 24 hours after ICU admission are screened for eligibility by the ICU physicians and clinical research nurses, around the clock and 7 days per week.

### Randomization

Consecutive eligible patients are randomly allocated in a 1:1 ratio to one of the two treatment groups: the EN group or the PN group. Randomization is stratified by centers. The patients are enrolled in each ICU by the local physicians and a clinical research nurse and/or clinical research assistant. Randomization and concealment are ensured by using a secure, computer-generated, interactive, response system available at each study center and managed by the biometrical unit of the Tours University Hospital, which has no role in recruitment.

### Study interventions

**Figure 1:**
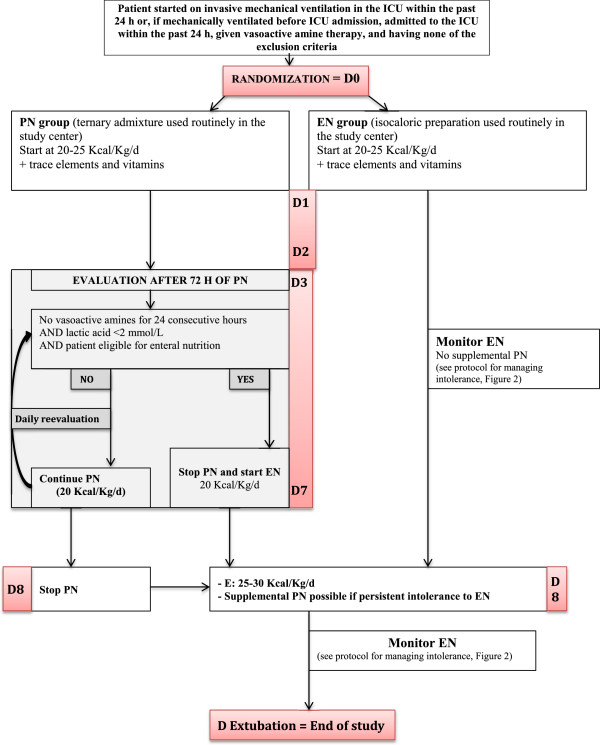
**Study protocol.** D, Day; EN, Enteral nutrition; ICU, Intensive care unit; PN, Parenteral nutrition.

The study protocol and randomization arms are detailed in Figure 1.

#### Parenteral nutrition group

In the PN group, the patients receive first-line PN for at least 72 hours. The route used subsequently depends on the results of the daily hemodynamic evaluations. If the hemodynamic condition is stable (no vasoactive drugs for 24 consecutive hours and arterial lactic acid level below 2 mmol/L) and the enteral route can be used, PN is stopped and immediately replaced by EN at the flow rate needed to achieve the previously defined calorie target. If, on the contrary, the patient still requires vasoactive drugs and/or the arterial lactic acid level is equal to or greater than 2 mmol/L, PN is continued for a total of 7 days (168 hours). On day 8, in the absence of contraindications to EN, PN is stopped and EN started, regardless of hemodynamic status. Supplemental PN may be added in the event of persistent intolerance to EN precluding achievement of the predefined calorie target.

#### Enteral nutrition group

In the EN group, the patients receive first-line EN. In the event of persistent gastrointestinal intolerance precluding achievement of the predefined calorie targets, supplemental PN may be added starting on day 8 [3, 62]. Isosmotic, isocaloric, normal protein polymeric preparations are used during the first week, after which the choice of the preparation is at the discretion of the physician at the bedside. To minimize the risk of upper gastrointestinal intolerance and consequently of vomiting, the volume of supplemental water given via the gastric route is as small as possible during the first study week.

### Nutritional support protocol

The nutritional support protocol, including measures designed to evaluate tolerance, is standardized as indicated below.

#### General principles of nutritional support in both study arms

Nutritional support is started as soon as possible after IMV initiation and no later than 24 hours after intubation or after ICU admission in patients intubated before ICU admission. The calorie target for each patient is estimated based on body weight as 20 to 25 kcal/kg/day during the acute phase (day 0 to day 7), then 25–30 kcal/kg/day from day 8 to extubation. The recommended calorie target is 20 kcal/kg/day during the acute phase (day 0 to day 7) and 30 kcal/kg/day from day 8 to extubation. In obese patients (body mass index (BMI) greater than 30 kg/m^2^), the body weight yielding a BMI of 30 kg/m^2^ is used to estimate the calorie target.

Nutritional support is prescribed as a flow rate (in milliliters per hour) and started at the flow rate required to achieve the calorie target on day 1 (as opposed to a gradual increase). The feed is delivered continuously over the 24-hour cycle without interruption. Actual feed delivery is monitored regularly by comparing delivered volumes to predefined daily calorie targets. In addition, special attention is directed to avoiding delays. Any interruption in feed delivery is reported to the ICU physician in charge. Usually, nutritional support is not interrupted while transporting the patient. However, when EN or PN must be interrupted (for example, for a specific gastrointestinal or radiological investigation), the flow rate is not increased to compensate for the interruption. All patients are kept in the semirecumbent supine position.

After extubation, regardless of time since randomization, decisions about the continued need for, and optimal route of, nutritional support are made by the physician in charge of the patient. Patients who are reintubated within 7 days after trial inclusion are managed until the end of the acute phase according to the arm to which they were randomized during the first period of intubation. Patients reintubated after the end of the acute phase (day 7) receive EN in the absence of contraindications.

#### Enteral nutrition

The feed is delivered continuously via a 14-French silicone gastric tube. The tube’s position in the middle of the stomach is checked on a radiograph obtained at ICU admission or immediately after tube placement, as well as when the tube is changed or repositioned. Special attention is directed to the risk of tube obstruction, particularly when administering medications. The tube is flushed regularly with 20 to 30 ml of water.

**Figure 2:**
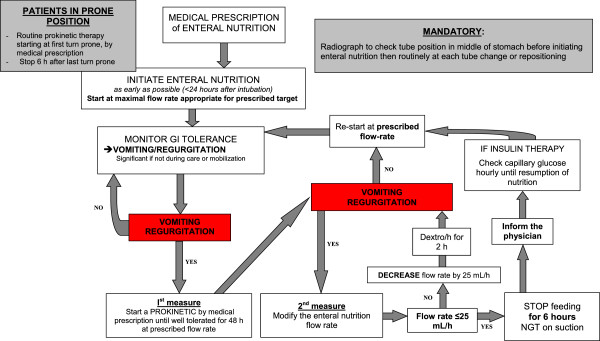
**Protocol for managing upper gastrointestinal intolerance.** GI, Gastrointestinal; NGT, Nasogastric tube. Response J Reignier: this is correct.

A predefined protocol is used to manage upper gastrointestinal intolerance to EN (Figure 2). Residual gastric volume is not monitored [63]. EN tolerance is assessed based only on episodes of significant vomiting or regurgitation (passage of feed into the mouth, outside the mouth or into the endotracheal tube in the absence of care procedures or mobilization). Minimal regurgitation or vomiting triggered by tracheal aspiration or oral cavity care is not taken to indicate intolerance. EN intolerance (that is, significant vomiting or regurgitation) leads to the following two measures. First, treatment with a prokinetic agent is to be administered after confirmation that there are no contraindications. The study ICUs use the prokinetic agent of their choice according to their standard practice. The prokinetic agent is continued until EN at the highest prescribed flow rate has been well tolerated for 48 hours. The prokinetic agent is then discontinued. Second, if gastric intolerance persists despite prokinetic therapy, the flow rate is decreased by 25 ml/hr every 6 hours until the signs of intolerance resolve. Therefore, EN is stopped (and the gastric tube placed under suction) only in patients with intolerance despite a flow rate ≤25 ml/hr. All interruptions in EN delivery must be reported to the physician in charge of the patient. EN is resumed at the prescribed flow rate (appropriate to the patient’s needs) after 6 hours have elapsed without further signs of intolerance. Patients at high risk for gastric intolerance, such as those turned in the prone position for acute respiratory distress syndrome, receive prophylactic prokinetic treatment starting at the first turn in the prone position [64, 65].

#### Parenteral nutrition

Ternary admixtures packaged in bags and containing the three groups of macronutrients are used according to standard practice in each participating center. Supplemental electrolytes are provided in a solution separate from the parenteral feed, according to the needs of each patient. PN is delivered continuously via a central venous catheter (CVC). Special attention is directed to preventing infections by complying with the standard protocols for CVC insertion and maintenance followed in each of the participating centers. Proper CVC position is checked routinely on a radiograph.

#### Additional intakes

Additional water and electrolytes are given intravenously according to the needs of each patient as assessed by the physician in charge and in compliance with standard practice in each study ICU. Intravenous vitamins and trace elements are given according to the needs of each individual patient using the standard preparations and protocols available in each study ICU. These components are not added to the PN bags. Instead, they are given continuously over the 24-hour cycle using a separate intravenous bag and line; if needed, this separate preparation is shielded from light using aluminum foil.

#### Intestinal transit monitoring

**Figure 3:**
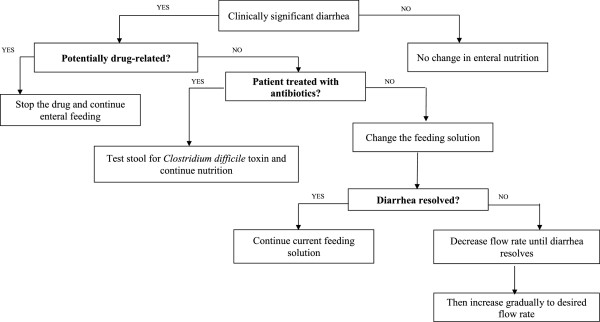
**Protocol for diarrhea management.**

Stool volume and appearance are monitored daily. Constipation (no stool for more than 6 days) or diarrhea (more than 300 ml of liquid stool or more than four loose stools per day) must be reported. Episodes of diarrhea are managed according to a specific diagnostic and therapeutic protocol (Figure 3) [66].

### Diagnosis of ventilator-associated pneumonia

VAP is suspected in patients with new and persistent or progressive lung infiltrates on the chest radiograph, combined with at least two of the following criteria: body temperature ≥38.5°C or ≤35.5°C, peripheral leukocytosis (>10,000/mm^3^) or leukopenia (<4,000/mm^3^), and purulent tracheal aspirates. The diagnosis must be confirmed in each participating ICU on the basis of a positive semiquantitative bacteriological result from a distal respiratory specimen: bronchoalveolar lavage fluid (positive if there are ≥10^4^ colony-forming units (cfu)/ml), protected specimen brush (positive if there are ≥10^3^ cfu/ml) or tracheobronchial aspirate (positive if there are ≥10^5^ cfu/ml) [29, 67]. VAP episodes are recorded from 48 hours after intubation until day 2 after extubation.

### Diagnosis of bowel ischemia

*Bowel ischemia* is defined in this trial as absent blood flow in one of the main arteries supplying the bowel (superior mesenteric artery, inferior mesenteric artery or celiac artery) with evidence of bowel wall compromise on an imaging study (computed tomography angiography, angiography or magnetic resonance angiography) or the presence of rectosigmoidoscopy- or colonoscopy-based criteria for colonic ischemia according to the Favier classification system (stage I, petechiae; stage II, petechiae and superficial ulcers; and stage III, necrotic ulcers and polypoid lesions) [68].

### Data collection and follow-up

At the time of inclusion, the following baseline characteristics are recorded: age, sex, date of ICU admission, height, body weight, BMI, primary diagnosis, McCabe score, Knaus score and preexisting comorbidities (including chronic renal failure, liver failure, pulmonary disease and heart failure; malignant disease; and immunosuppression). Use of sedatives, insulin, proton pump inhibitors, dialysis, neuromuscular blockers and gastric prokinetic agents before inclusion are also recorded. The Simplified Acute Physiology Score II is computed 24 hours after ICU admission [69].

The information listed below is recorded daily until extubation or until day 28 during intubation, whichever occurs first.

1. *Nutritional data*: name of the enteral or parenteral preparation, target volume and number of calories, volume and calories delivered per day, vomiting (yes/no, daily), decrease or discontinuation of nutritional support (yes/no), reason for decreasing or discontinuing nutritional support (vomiting, diarrhea, acute colonic pseudo-obstruction, other acute abdominal symptoms, abdominal complication, imaging study, other), stools (yes/no, daily) and diarrhea with its presumed cause (intolerance to nutritional support, medication, *Clostridium difficile*, other).
2. *Treatments*: prokinetic agent, sedation, neuromuscular blockers, catecholamines (dobutamine, noradrenaline or adrenaline), renal replacement therapy, antibiotics, insulin (total dose/24 hr), volume of intravenous fluids (total/24 hr) and gastric antisecretory agents (sucralfate, proton pump inhibitors, histamine receptor antagonists, other).
3. *Laboratory data*: (a) at baseline—hemoglobin, leukocytes, platelets, Na^+^, K^+^, Ca^2+^, pH, triglycerides, partial pressure of oxygen in arterial blood (PaO_2_), partial pressure of carbon dioxide in arterial blood, pH, arterial lactic acid, bicarbonate, urea, creatinine, bilirubin, aspartate aminotransferase (AST), alanine aminotransferase (ALT), γ-glutamyl transpeptidase, alkaline phosphatase and total protein; (b) daily from day 1 to day 7 or until extubation—PaO_2_, arterial lactic acid, bilirubin, AST, ALT, γ-glutamyl transpeptidase, alkaline phosphatase, glucose, protein, Na^+^, K^+^, Ca^2+^, pH, triglycerides and creatinine; (c) on day 1 and on day 7 or the day of extubation—albumin, prealbumin and C-reactive protein levels; daily from day 1 to day 7—Sepsis-related Organ Failure Assessment (SOFA) score [70].
4. *Nosocomial infections* (one data collection form per infection): VAP (date of diagnosis, organism, resistance profile), bacteremia (date of diagnosis, organism, resistance profile), intravascular catheter-related infection (date of diagnosis, organism, resistance profile), urinary tract infection (date of diagnosis, organism, resistance profile), soft tissue infection (date of diagnosis, organism, resistance profile) and other (type, date of diagnosis, organism, resistance profile).
5. *Invasive devices*: endotracheal tube, intravascular catheters and urinary catheters, with the dates of insertion and removal for each.
6. Each patient is followed until hospital discharge or day 90, whichever occurs first. Vital status is recorded at ICU discharge, at hospital discharge, on day 28 and on day 90. Table  1 is the study flowchart.

**Table 1:** **Study flowchart**

	Inclusion	Day 0 ^a^	Day 1 to Day ***n***	End of study	Day 28	Day 90
Eligibility: check inclusion and exclusion criteria	X					
Patient information and consent	X					
Randomization	X					
Demographics	X					
Characteristics		X				
Ventilation		X				
Laboratory tests		X	X			
SOFA score^b^		X	X			
Nutritional evaluation		X	X			
Treatments used		X	X			
Daily calorie intake			X			
Nosocomial infections			X			
Final extubation				X		
Final discontinuation of nutritional support				X		
Survived/died				X	X	X

^a^From time at inclusion to 11:59 pm. ^b^SOFA, Sepsis-related Organ Failure Assessment.

### Organization of the trial

#### Funding/support

NUTRIREA-2 is sponsored by the La Roche sur Yon Hospital (Centre Hospitalier de la Vendée, La Roche sur Yon, France) and supported by a grant from the French Ministry of Health (Programme Hospitalier de Recherche Clinique 2012, PHRC-12-0184).

#### Coordination and conduct of the trial

Before the start of patient recruitment procedures, all physicians and other health-care workers in the 44 participating ICUs attended formal training sessions on the study protocol and data collection in the electronic case report form (eCRF). All documents required for the study are available in each ICU. The eCRF is a secure, interactive, web response system available at each study center, provided and managed by the biometrical unit of the Tours University Hospital (CIC INSERM 1415, Tours, France). In each participating ICU, the physicians and a clinical research nurse and/or clinical research assistant are in charge of daily patient screening and inclusion, ensuring compliance with the study protocol and collecting the study data in the eCRFs. The Clinical Research Unit of the La Roche-sur-Yon Hospital will review the screening forms and clinical data at regular intervals. The principal investigators will meet with the ICU teams to discuss any problems with data collection and protocol compliance and to evaluate study progress. According to French law, the eCRF and database organization have been approved by the appropriate committees (CCTIRS: Comité Consultatif sur le Traitement de l’Information en matière de Recherche dans le domaine de la Santé; and CNIL: Commission Nationale de l’Informatique et des Libertés).

#### Interim analyses

Given the need for a large sample size, two interim analyses are scheduled, one after enrollment of 1,000 patients and the other after enrollment of 2,000 patients. The independent Data Safety Monitoring Board (DSMB) is composed of two physicians and one biostatistician not otherwise involved in the trial. For both interim analyses, the DSMB will have access to unblinded results on day 28 mortality, variations in SOFA scores from day 1 to day 7, blood bilirubin values and nosocomial infections. The results of the interim analyses will not be disclosed unless they lead the DSMB to request premature trial discontinuation.

### Blinding

Blinding of the physicians, nurses and patients to the use of EN and/or PN is not feasible. The absence of blinding cannot have an impact, however, because the primary outcome is objective (day 28 mortality) [71]. Moreover, all VAP diagnoses are adjudicated by an independent blinded committee on the basis of all available clinical, radiological and bacteriological data.

### Study outcomes

#### Primary endpoint

The primary endpoint is all-cause mortality by day 28. Information on this endpoint is collected on the 28th day after patient inclusion in the study. For discharged patients, information on the primary endpoint is collected by a telephone call to the patient at home.

#### Secondary endpoints

The following are the secondary endpoints of the study:

1. The proportion of patients with at least one VAP episode
2. VAP incidence density per 1,000 days of IMV (that is, ratio of the number of patients with at least one VAP episode during IMV or within 2 days after extubation over the period at risk, defined as follows: total number of IMV days in patients without VAP or number of IMV days before the first VAP episode in patients with VAP)
3. Number of VAP episodes per patient
4. Proportion of patients with at least one episode of bacteremia and incidence density of bacteremia per 1,000 ICU days
5. Proportion of patients with at least one CVC-related infection and incidence density of CVC-related infections per 1,000 CVC days
6. Proportion of patients with at least one episode of urinary tract infection and incidence density of urinary tract infections per 1,000 urinary catheter days
7. Proportion of patients with at least one soft tissue infection and incidence density of soft tissue infections per 1,000 ICU days
8. Proportion of patients with other nosocomial infections and incidence density of other nosocomial infections per 1,000 ICU days
9. Proportion of patients with at least one nosocomial infection
10. Descriptive bacteriological data (organisms recovered in the overall population of nosocomial infections and antimicrobial resistance profiles)
11. Proportion of patients with at least one episode of vomiting or regurgitation during IMV
12. Proportion of patients with at least one episode of diarrhea
13. Proportion of patients with at least one documented episode of bowel ischemia
14. Number of calories (in kilocalories) delivered enterally and parenterally (daily mean during the first week and daily mean throughout the time on IMV)
15. Ratio (as a percentage) of prescribed over delivered calories via the enteral and parenteral routes (daily mean during the first week, daily mean throughout the time on IMV and proportion of patients who achieved their calorie target on each follow-up day)
16. Volume of liquid feed (in milliliters) delivered (daily mean during the first week and daily mean throughout the time on IMV)
17. Variations in SOFA scores from day 1 to day 7
18. ICU mortality, 90-day mortality and hospital mortality
19. Mean changes in serum albumin, prealbumin and C-reactive protein measured at baseline, at the end of IMV, on day 7 (in patients on IMV for more than 7 days) and at ICU discharge
20. Proportion of patients with at least one liver dysfunction episode (defined as serum bilirubin >50 μmol/L and/or γ-glutamyl transferase, alkaline phosphatase and/or AST or ALT >3 N) evaluated at the end of IMV, on day 7 (in patients on IMV for more than 7 days) and at ICU discharge
21. ICU length of stay
22. Hospital length of stay
23. Time on IMV
24. Changes in mean body weight determined at baseline, on day 7 and at ICU discharge

### Sample size

The working hypothesis of this randomized controlled trial is that day 28 all-cause mortality in patients admitted to the ICU and treated with IMV and vasoactive drugs will be lower when early nutritional support is provided enterally rather than parenterally. We have determined that 1,427 patients are needed in each group (2,854 patients in all), assuming a 37% day 28 mortality rate in the PN group and a 5% decrease in mortality with early EN (that is, a 32% day 28 mortality rate), with a 4.9% two-sided type I error rate and 80% power, given that two interim analyses will be performed, and given that the statistical tests will be performed using significance thresholds of 0.001 for the two interim analyses and 0.049 for the final analysis according to the Peto’s method [72]. The mortality rates used for the sample size estimation are those obtained in the NUTRIREA-1 randomized controlled trial performed in nine centers using similar inclusion criteria [63].

### Statistical analysis

**Figure 4:**
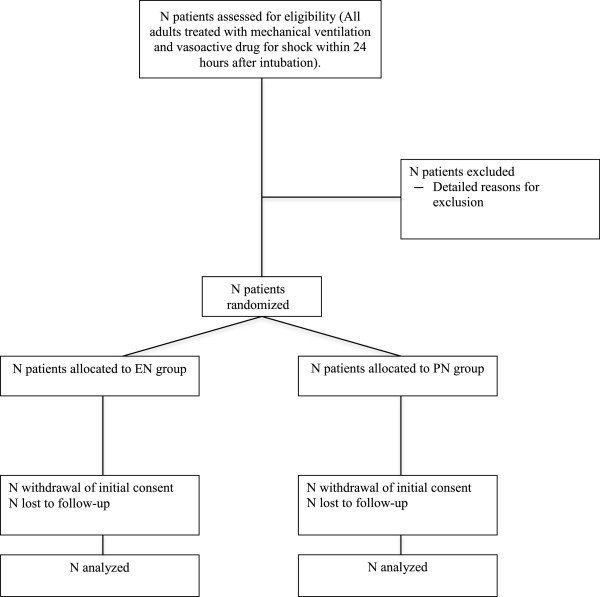
**Flow diagram of NUTRIREA-2 trial according to CONSORT.**

The statistical analysis will follow the intention-to-treat approach; that is, each patient will remain in the group assigned by randomization, regardless of subsequent events. A statistical analysis report will be written to describe all the findings according to CONSORT statement recommendations, taking into account the specific features of the trial, most notably the nonpharmacological nature of the intervention (Figure 4). The baseline features of the groups established by randomization will be compared using descriptive statistics. No statistical tests will be performed.

#### Primary endpoint

Day 28 mortality will be reported as the point estimates with the 95% confidence intervals in each group and compared between the two groups using the χ^2^ test.

#### Secondary endpoints

The analysis of patients with at least one nosocomial infection will rely on the competing-risk concept with death as the competing risk, because patients who have died cannot experience nosocomial infections. For the proportion of patients with at least one VAP episode, the method used will be the same as that used for nosocomial infections, with not only death as a competing risk but also time to extubation +2 days, because, after this point, any episode of pneumonia would not be classified as a VAP episode. The number of VAP episodes per patient will be analyzed using a negative binomial regression model with no offset variable. Bacteremia, CVC-related infections, urinary tract infections and soft tissue infections will be analyzed using the same method as that used for the pooled nosocomial infections. For descriptive bacteriological data (organisms recovered with their resistance profiles for each nosocomial infection), only descriptive analyses will be performed. For the proportion of patients with at least one episode of vomiting or regurgitation, diarrhea, constipation, documented acute colonic pseudo-obstruction (Ogilvie syndrome), documented bowel ischemia, mechanical complication of CVC insertion and liver dysfunction, the method used will be the same as that employed for the nosocomial infections. Changes over time in calories delivered daily by the enteral and parenteral routes and changes over time in enteral and parenteral feed volumes delivered daily will be compared between the two groups using a mixed linear model, after data transformation if necessary. The proportions of patients who achieved their daily calorie target will be compared between the two groups using a logistic random-effects model. Day 90 mortality will be analyzed in the same way as day 28 mortality. For ICU and hospital mortality rates, competing-risk models will be used, as ICU discharge and hospital discharge compete with death during the stay. Changes over time in nutritional markers and body weight will be analyzed using the method described above for the number of calories delivered. ICU length of stay, hospital length of stay and IMV duration will be compared between the two groups using nonparametric Wilcoxon tests.

## Discussion

Nutritional support, whether delivered enterally or parenterally, is among the first-line treatments used routinely in the ICU. Guidelines state that the enteral route should be preferred to the parenteral route and should be used early in the course of the critical illness. However, guidelines are poorly followed and are not supported by adequately designed clinical studies. To date, no study has produced definitive information on the risks and benefits of EN versus PN in critical care. NUTRIREA-2 is the first large randomized controlled trial designed to evaluate the hypothesis that early EN decreases mortality compared to PN in ICU patients. The NUTRIREA-2 sample size provides sufficient statistical power to detect a significant mortality rate decrease on day 28. The study focuses on patients receiving IMV and vasoactive drugs for shock, because such very severely ill ICU patients with multiple organ failure may be most likely to benefit from early EN compared to early PN. Furthermore, at present, many of these patients do not receive early nutritional support or early PN, despite being eligible for EN. However, these patients are considered at high risk for complications of EN, such as upper gastrointestinal intolerance, VAP or bowel ischemia. If the study hypothesis is confirmed, early EN will become the reference standard for initial nutritional support in patients with shock who are receiving IMV, and this change will decrease morbidity and mortality rates and improve patient outcomes.

## Trial status

Enrollment is ongoing. Inclusion started in March 2013. By 10 March 2014, 1,000 patients had been included. The first interim analysis was performed on these 1,000 patients and led the DSMB to recommend continuation of the study. By 15 December 2014, 1,795 patients had been included. Recruitment is expected to be complete by November 2015.

## Abbreviations

ALT: Alanine aminotransferase
AST: Aspartate aminotransferase
BMI: Body mass index
CCTIRS: Comité Consultatif sur le Traitement de l’Information en matière de Recherche dans le domaine de la Santé (French Advisory Committee on Healthcare Research Data Handling)
CépiDc: Centre d’épidémiologie sur les causes médicales de décès (Epidemiology Center for Causes of Death)
CNIL: Commission Nationale de l’Informatique et des Libertés (French Data Protection Authority)
CVC: Central venous catheter
DSMB: Data Safety Monitoring Board
eCRF: Electronic case report form
EN: Enteral nutrition
ICU: intensive care unit
PN: Parenteral nutrition
SOFA: Sepsis-related Organ Failure Assessment
SRLF: Société de Réanimation de Langue Française (French-Speaking Society for Critical Care)
VAP: Ventilator-associated pneumonia.
